# Heads or tails? Differential translational regulation in cercarial heads and tails of schistosome worms

**DOI:** 10.1371/journal.pone.0224358

**Published:** 2019-10-28

**Authors:** James R. Hagerty, Emmitt R. Jolly

**Affiliations:** 1 Case Western Reserve University, Department of Biology, Cleveland, OH, United States of America; 2 Case Western Reserve University, Center for Global Health and Disease, Cleveland, OH, United States of America; Alexandria University, EGYPT

## Abstract

Schistosomes are obligate helminths responsible for over 218 million cases of human schistosomiasis in 78 countries around the world. Infection occurs when free-swimming cercariae penetrate human skin and initiate developmental progression into parasitic obligate worms that consume red blood cells. Transcriptomic studies of infectious cercariae reveal abundant mRNAs associated with energy metabolism and host invasion. However, the cercaria is mostly transcriptionally quiescent, suggesting that most mRNAs are primed prior to cercarial escape from the snail host. The use of transcriptomics to understand protein expression presumes that transcription and translation are functionally coupled and the cercarial stage has categorically been treated as a single unit for -omic analysis. Per contra, the relationship between transcription and translation in infectious cercariae has not been described. To understand the correlation between transcription and translation in cercariae, we separately measured nascent translation levels in cercarial heads, cercarial tails and in the developing schistosomula, the next stage of its life cycle. The loss of the cercarial tail is essential for the transformation from a cercaria to a schistosomulum. We observed that translation was initially limited and the translation rate accelerated during the first 72-hours after tail loss. When we tested nascent translation in cercarial heads, cercarial tails, whole cercariae, and 4-hour schistosomula, we found that translation is significantly upregulated in the cercarial tail when compared to the cercarial head and that translation was undetectable in heads using immunofluorescent image quantification (p = .0005). These data represent a major shift in how we understand the cercarial stage. The cercarial head is mostly transcriptionally and translationally quiescent while being sufficient for progression into a schistosomulum. In addition, transcription and translation are not linked in *Schistosoma mansoni* cercaria. Thus, our current conceptual approach of treating the cercaria as a single functional unit for -omic studies may be insufficient to understand cercarial development.

## Introduction

Schistosomes have a complex lifecycle characterized by a striking series of morphological and developmental transitions between an invertebrate host and a definitive human host, with two intermediate free- swimming stages. Adult schistosomes reside in the mesentery of the human liver or bladder, depending on the species, where they pair, mate, and produce hundreds of eggs daily. Some of the eggs are excreted from the human host into fresh water where they hatch into free-swimming miracidia that infect a molluscan snail host. After infecting the snail, the miracidia metamorphose into sporocysts that produce free-swimming and transient cercariae that must find a human host for continued survival. The cercariae penetrate host skin, losing the cercarial tail during penetration, leaving only the cercarial head that transforms into a schistosomulum.

The cercaria to schistosomulum transition involves a quick series of adaptive responses following divestiture of the cercarial tail. Within 1 hour these responses include the loss of the cercarial glycocalyx, the conversion of outer tegument from a 3-layered to a 7-layered structure, the shift in energy production from aerobic to anaerobic, and a new schistosomulum that can no longer tolerate fresh water but is adapted for the host’s saline environment [1,2]. The schistosomulum initiates expansion of a rudimentary gut for digestion of red blood cells, produces new muscle proteins, reorganizes its nervous system, and evades the host immune system, eventually developing into a sexually mature adult worm over a 6–7 week period *in vivo* [3,4].

The motile and infectious cercaria is transcriptionally silent [5]. This transcriptional repression is de-repressed after the cercaria enters the mammalian host, loses its tail, and transforms into a schistosomulum. The schistosomulum then initiates a large burst of transcriptional activity within 3 hours after tail loss [6]. However, it is unclear whether cercariae similarly down regulate protein production, or whether transcription and translation are decoupled at this stage since cercariae are transcriptionally quiescent [5]. Blanton et al. suggested that translation appears to be blocked following the transformation from cercariae to schistosomula [7]. In addition, we previously observed that after transfection of schistosomula with heterologous expression plasmids, transcript levels of the heterologous reporter could easily be detected, but we detected very low protein levels [6]. The mechanisms used by cercaria and schistosomula to regulate translation and stabilize quiescent transcripts have not been fully described. The translational inhibitor puromycin has little effect on a cercarial transformation and development into later life stages [5]. The combination of these data led us to directly explore the levels of nascent translation in cercariae and in schistosomula.

In order to probe the levels of translation in schistosomes, we used puromycin, an aminoacyl-tRNA that covalently interacts with nascent peptide chains and can be detected using anti-puromycin antibodies. Puromycin truncates peptides that directly interact with the ribosome by entering the ribosomal A site, the point of entry for the aminoacyl-tRNA. Once the peptidyl transferase reaction has occurred, the puromycin molecule is unable to be cleaved and the peptide chain is released prematurely. The premature release caused by puromycin does not degrade or destroy the ribosome [8]. The anti-puromycin antibody can then be used for detection via western blot or immunohistochemistry. Both of these methods were utilized in this study. Anisomycin is another translational inhibitor that actively competes with puromycin for entry into the A site of the ribosome [9], and competition between the two inhibitors can be utilized to determine specificity of the interaction with puromycin.

In this report, we used immunohistochemistry and protein immunoblotting to quantitate levels of translation in cercariae and in schistosomula. Cercariae are made up of two macrostructures, the cercarial head and the cercarial tail. The cercarial tail, necessary for motility in water and invasion, is divested after penetration through host skin. The cercarial head contains all of the required components for development into a schistosomulum after tail divestiture. Thus, we separated cercariae into two macromolecular segments: cercarial heads and tails. We observed that translation in cercarial heads and tails are differentially regulated. In particular, cercarial tails have higher levels of translation activity and translation activity is extremely limited in cercarial heads. Finally, we found that, while translation in schistosomula is inhibited after transformation, translation levels slowly continue to increase over a 3 day period, a time when transcription levels are steadily high.

## Methods

### Parasite collection

*Biomphalaria glabrata* snails infected with S*chistosoma mansoni* (NMRI strain) were obtained from Biomedical Research Institute (BRI; Rockville, MD). Cercariae were shed as previously described [9].

### Head vs tail separation

Cercariae were mechanically transformed in incomplete DMEM (Gibco) by passing through 22-gauge needle 50 times. Cercarial heads were separated from cercarial tails using a 70% percoll gradient and centrifuged at 350 X g for 30 minutes at 4°C [10]. Cercarial head and tail fractions were collected separately and washed 3 times with incomplete DMEM. Subsequently, schistosomula were cultured in complete DMEM (5% FBS 1x Pen/Strep) at 37°C for 4 hours, collected and washed with PBS, and resuspended in protein buffer (25mM 7.5PH Tris, 1mM DTT, 2X Halt protease inhibitor (Thermo Scientific), 40U/mL RNaseOUT (Invitrogen), 20 mM PMSF). Frozen cercarial pellets for head and tail only protein extraction were thawed on ice in incomplete DMEM containing 2X HALT protease inhibitor. Live cercariae were shed as above and suspended in incomplete DMEM 2X Halt and 10% ethanol solution. Ethanol-killed cercariae were passed through 22-gauge needle 20 times and concentrated at 1000 X g for 30 seconds. The ethanol-killed cercariae were combined with the thawed cercariae samples. To isolate cercarial heads and tails, cercariae were separated by being passed through a double 22-gauge needle for 30 times and viewed under a low-mag scope to determine if heads and tails were separated or intact. The frozen cercarial samples where passed through the needle for 30 total passes to reduce damage of the more delicate thawed tissue. 3 mL of the DMEM-Halt and separated cercariae were then added to 10 mL of 70% Percoll gradient and centrifuged at 1000 X g for 25 minutes at 4C as previously described [10,11]. Cercarial heads and cercarial tails were separated into two fractions within the column, were removed and washed with incomplete DMEM 3 times, and then centrifuged at 1000g for 3 minutes. Two additional washes were used to clean intermediate fraction. Small samples were visualized to determine appropriate separation. Head samples showed a minimal amount of tail contamination. Tail samples showed no visible head contamination.

### Translational inhibitor-based swimming and longevity assay

Cercariae were cultured overnight at 16° C as previously described [12]. Translational inhibitors were added at the following concentrations: 455 μM, 910 μM, and 1365 μM puromycin [13] and 525 μM, 787 μM, and 1050 μM emetine [14], and observed at 4-hours, 8-hours, and 12-hours after the additions of drug treatment for the swimming assay. Approximately 75 cercariae per well were cultured in 9696-well plate and each treatment was administered in triplicate. All treatments including the Wild Type (WT) untreated control samples were performed in a 200 μL total volume of water. The longevity assay utilized anisomycin at 24 μM, 72 μM, 120 μM, and 240 μM and puromycin at 115 μM, 230 μM, 345 μM, and 460 μM. The individuals were observed at 24-hours and 48-hours for viability only. Propidium iodide stain was added at 2 μg/mL concentration as a lethality stain to quantify viable individuals [15]. Leica DMI8 10x objective was used to observe individuals under bright field and with the Texas red cube set for propidium iodide staining. Individuals showing any level of propidium iodide staining were considered non-viable. Images were captured at the focal plane of the bottom of each well at each time point each time point [16]. The individuals were then killed using iodine and counted to determine total population in each well. The arithmetic difference between the total number of individuals observed after iodine treatment and individuals settled at the bottom of the well gives the number of individuals swimming in the water column. (Iodine Killed Cercariae-Settled Cercariae = Swimming Cercariae). The significance of the data collected was calculated using one-way ANOVA with Dunnett’s multiple comparison against untreated samples.

### Anisomycin competition assay

Anisomycin (EMD Millipore) was added to 72-hour schistomula at a concentration of 455 μM and incubated at 37° C for 20 minutes. Puromycin was then added at 91 μM and samples were incubated for 30 minutes at 37° C. Subsequently, samples were fixed and processed for analysis by immunohistochemistry.

### Protein extraction

Protein extraction was performed as previously described with the following modifications: addition of 20 units per mL of RNAseOUT (Thermo Scientific) and required drug treatments [17]. All samples were incubated at 37° C for 1 hour to allow puromycin integration. One hundred μL of 425–600 μm glass beads (Sigma Aldrich) was added to each sample. Tubes were lab film wrapped and vortexed at full speed for ten 1 minute pulses with 1 minute on ice between pulses. Samples were centrifuged at 1000 X g for 2 minutes at 4° C and supernatant was added to 0.5 mL tubes. Samples were sonicated for five 30 second rounds with continuous power and incubated on ice for 1 minute between rounds. Samples were then centrifuged at 12000 X g for 15 minutes at 4° C to clarify solution. Protein quantitation was performed using BCA assay (Thermo Scientific) and measured on the ND-8000 (Thermo Scientific).

### Western blot

To detect nascent translation, 5.25 μg puromycin treated cercarial tail extract, 5.25 μg and 10 μg puromycin treated cercarial head, whole cercariae, and 4-hour schistosomula were resolved on an 8–18% polyacrylamide gel (GE Healthcare). 10 μg untreated whole cercarial and 4-hour schistosomula extract were used as a negative control. The gel was transferred to nitrocellulose membranes in ice-cold Towbin transfer solution (25 mM tris, 192 mM glycine, 20% methanol) at 400 mA for 1.5 hours. Following the transfer, the membranes were stained with 0.1% ponceau S for 5 minutes and rinsed with deionized water. The membrane was then blocked in 5% milk dissolved in phosphate buffered saline, 0.1% Tween-20 (PBSTw) on an orbital shaker at room temperature for 1 hour. Puromycin antibody (PUROMYCIN-2A4 DSHB) was added to a concentration of 1.66 μg/mL, and the membranes were gently rocked at 4°C overnight. The membranes were washed in PBSTw on an orbital shaker for 5, 10, and 15 minutes, after which an HRP-linked goat anti-mouse secondary antibody (GE Healthcare) was added at a dilution of 1:10,000 in 1% filtered milk/PBSTw, followed by orbital shaking at room temperature for 1 hour and washing in PBSTw. Amersham ECL Western blotting detection reagent (GE Healthcare) was added (2 mL per nitrocellulose membrane) and incubated at room temperature for 1 minute before the membranes were exposed to autoradiography film.

### Immunohistochemistry

Puromycin-treated cercariae, 24-hour, 48-hour, 72-hour schistosomula and untreated individuals from each stage were assessed by immunohistochemistry and prepared as previously described [18,19]. Where anisomycin is used as a competition to puromycin, samples were pretreated with anisomycin prior to puromycin treatment and fixation. Briefly, all samples were fixed for 20 minutes at room temperature in a 4% paraformaldehyde/PBSTw (PBS/0.1% Tween-20) solution, washed in PBSTw, then dehydrated in a methanol/PBSTw series and stored in 100% methanol at −20° C until use. Prior to use, cercariae were rehydrated, digested for 10 minutes at room temperature in permeabilization solution (1×PBSTw, 0.1% SDS, and proteinase K (1 μg/mL)), and washed in PBSTw (all subsequent washes were carried out with nutation at room temperature). All samples were re-fixed for 10 minutes at room temperature in a 4% paraformaldehyde/PBSTw solution, and washed in PBSTw. Samples were incubated with rocking in block solution (PBSTw, 5% horse serum (Jackson ImmunoResearch Laboratories), 0.05% Tween-20, and 0.3% Triton X-100) for 2 hours at room temperature or overnight at 4° C. The samples were then incubated with anti-puromycin antibody (PUROMYCIN-2A4 DSHB) in block solution at a concentration of 3.36 μg/mL overnight at 4° C and followed by a >2 hour wash at room temperature. Samples were then incubated with an Alexa 647 donkey anti-mouse antibody (Jackson ImmunoResearch Laboratories) at a concentration of 1:400 in block solution overnight at 4° C and washed in PBSTw (>2 hours.), at room temperature with the second wash containing DAPI (1 μg/mL). After washing, cercariae samples were mounted in Slow Fade Gold (Invitrogen). Schistosomula samples were mounted in Prolong Glass (Invitrogen).

### Confocal imaging

All cercarial images were taken using Leica SP8 STED confocal system with a tunable white light laser. The Alexa 647 conjugated secondary was excited using 647nm light from the tunable white light laser. DAPI stain was excited at 405nm using the UV diode. All schistosomula images were taken using the Leica SP8 confocal system with a 405nm UV diode for DAPI excitation and 633nm HeNe fixed laser for Alexa 647 conjugated secondary excitation. Detection of both DAPI and Alexa 647 for all images was set to peak absorbance using tunable hybrid detectors. All images were taken using an APO 63x/1.4 oil objective lens from Leica, analyzed and processed using Image J and Leica LASX software.

### Image quantification

Treated and untreated cercariae images were quantified using Image J. For all samples, max projections of DAPI signal and puromycin signal were averaged over manually drawn regions of interest (ROI’s) of head only and tail only. ROI selection was based on bright field channel to avoid potential debris and to offer complete coverage. The average 8-bit signal intensity for puromycin was normalized based on average DAPI intensity over the same region. Untreated samples were used to correct for background detection levels and the average normalized signal from head only and tail only was then removed from all treated head only and tail only signal quantification respectively. After background reduction heads only signal was set to 0. All samples were imaged using the same scope settings including laser intensity, gain, offset, gating, and pinhole. The P-value measuring the statistical differences between heads and tails was calculated the Mann-Whitney and an unpaired t-test.

## Results

### Cercarial heads and tails show differential translational regulation

It is generally accepted that the function of cercariae is to transfer genetic material to the next developmental stage and that cercarial functional transcripts revolved around energy metabolism for motility and host identification, which correlated with its biological function [4, 11, 20]. In continued support of this idea, it was recently reported that cercariae do not actively transcribe new RNAs, but maintain the initial subset of RNAs established prior to exit from the molluscan host [5]. This transcription inhibition is relieved after invasion in the mammalian host. However, translation levels are initially limited in the subsequent schistosomula stage [7]. Since cercariae do not undergo transcription, and the cercarial heads that transform into schistosomula, eventually becoming the adult worm, have limited translation after host invasion, we assessed whether there are differences in translation between cercarial heads and cercarial tails.

To measure translation in heads and tails, we adapted SunSet and Punch-P puromycylation approaches for use with live and lysed schistosomes. Puromycin is a structural analog of aminoacyl-tRNAs that can incorporate into nascent polypeptide chains and inhibit elongation of actively translating peptides by covalently associating with the nascent chain within the ribosome [21]. With equal affinity, puromycin can incorporate into nascent chains of lysed cell extract and the approach is as sensitive as RiboSeq [22]. Thus, newly translated proteins can be visualized after puromycin-labeling with antibodies against puromycin (2A4 DSHB) [20, 23]. This approach has been tested on multiple tissue types as well as cell lysates [21–24].

**Fig 1:**
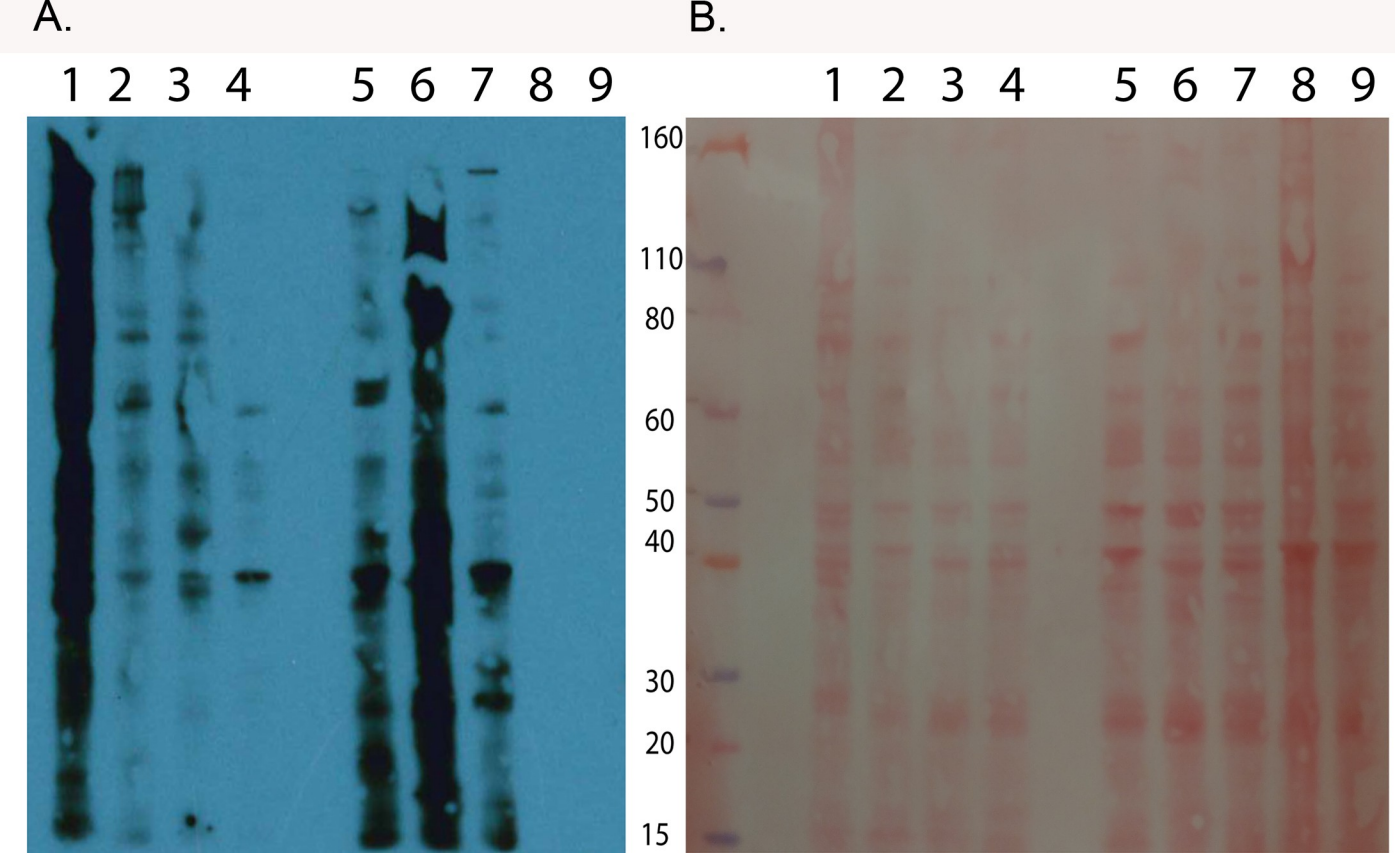
Nascent translation is repressed in early schistosomula and cercarial heads compared to cercarial tails. (A) An anti-puromycin PMY-2A4 was used to test for translation levels in puromycin treated (Lanes 1–7) and untreated (Lanes 8–9) samples. Lanes 1–4 have 5μg of total protein, and Lanes 5–9 have 10μg of total protein. Lane 1-cercarial tails only, lane 2-cercarial heads only, lane 3-whole cercariae, and lane 4 4-hour schistosomula. Lane 5-cercarial heads only, lane 6-whole cercariae, lane 7-4-hour schistosomula, lane 8-untreated whole cercariae, and lane 9-untreated 4-hour schistosomula. (B) Ponceau staining on the Western membrane to verify even protein concentration. The lane order is the same.

We puromycylated lysed cercarial heads and tails and were able to observe significant integration of puromycin by Western blot analysis (Fig 1). We found that cercarial tails (Fig 1, Lane 1) had significantly more active translation than cercarial heads and whole cercariae (Fig 1, Lanes 2 & 3 respectively). When we increased the concentration from 5μg to 10μg of protein, whole cercariae (Fig 1, Lane 6) had more translation than did heads (Fig 1, Lane 5), or 4 hour schistosomula (Fig 1, Lane 7) at the same protein concentration. These data suggest that active translation is limited in the initial 4 hours after cercariae have transformed to post-invasion schistosomula. We visualized total protein levels using Ponceau stain to ensure that protein levels were consistent between lanes (Fig 1).

**Fig 2:**
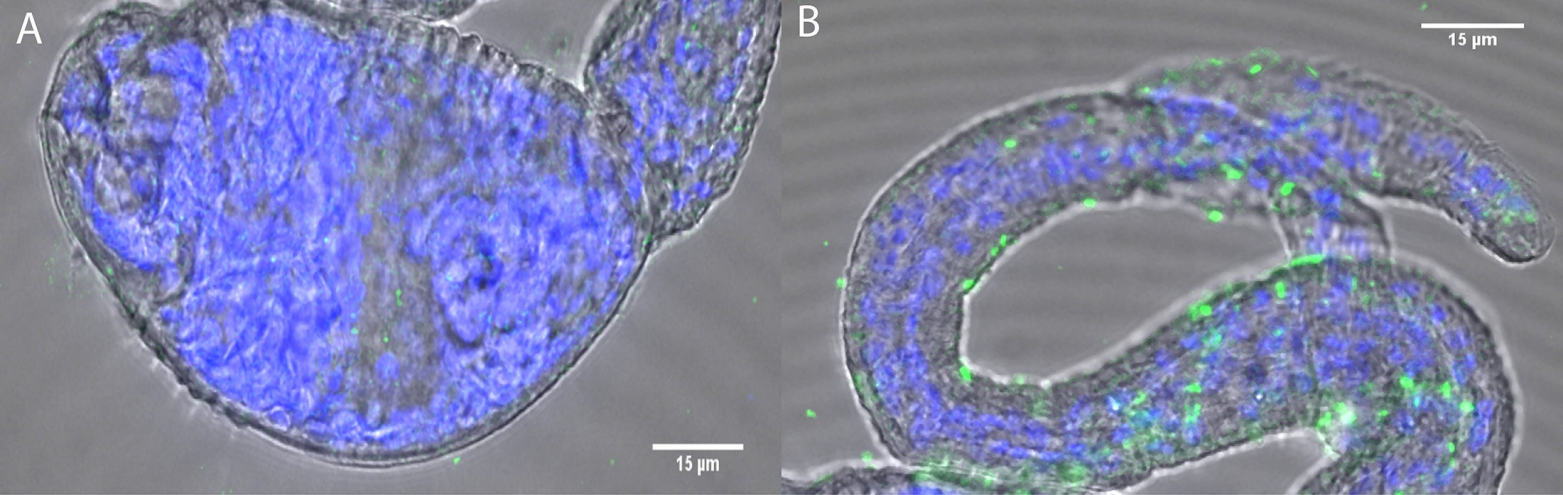
Nascent translation is repressed in heads compared to tails of cercarial *S*. *mansoni*. Representative confocal maximum projections of treated cercarial head and tail. Anti-Puromycin PMY-2A4 primary with Alexa 647 secondary was used to detect nascent translation in cercariae. Head (A) and Tail (B) regions were stained for DAPI (blue) and PMY (green) after background signal reduction using untreated samples. (A) shows little detectable nascent translation signal. (B) shows nascent translation throughout the tail region. Background correction was done using Image J.

**Fig 3:**
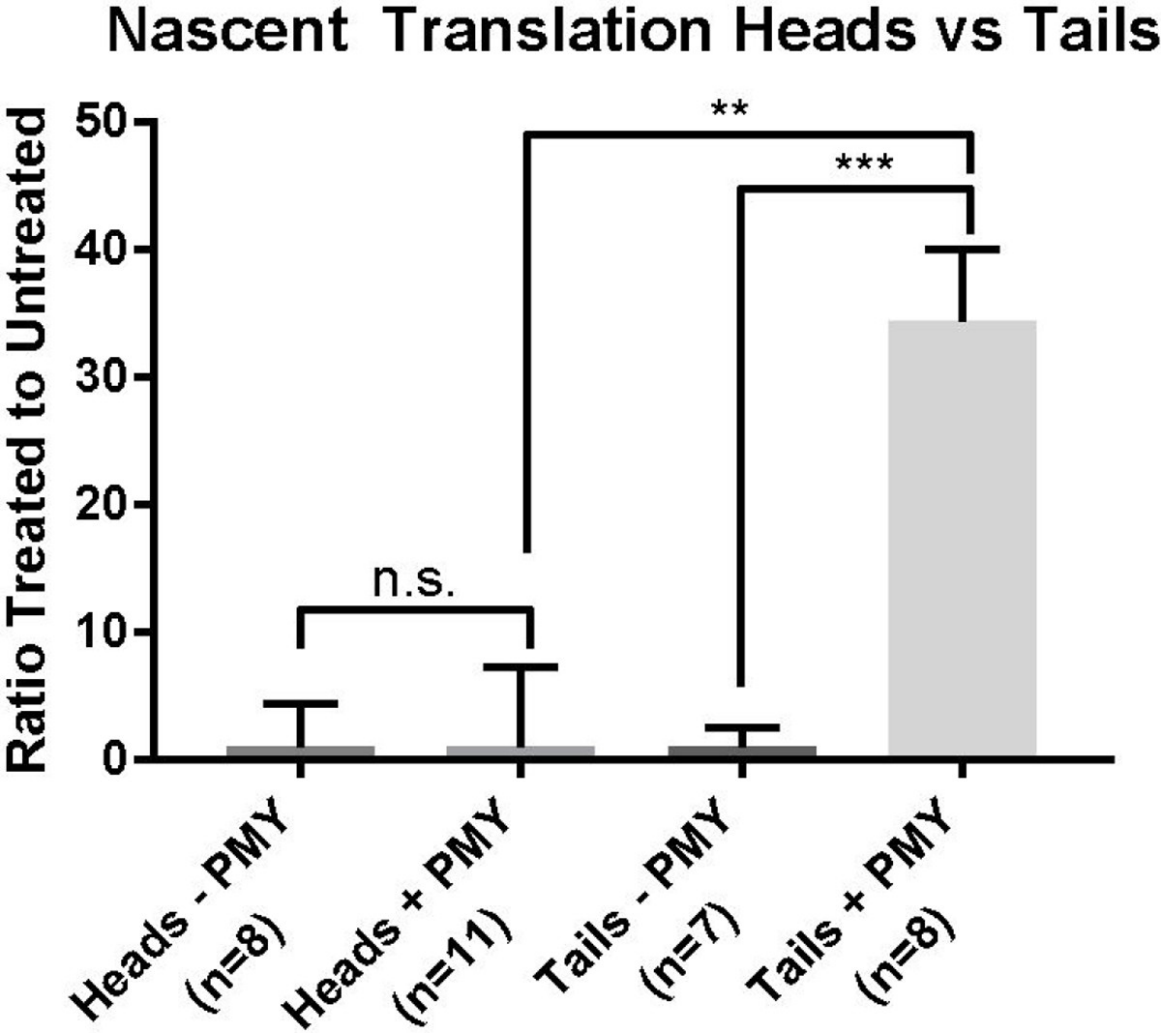
Quantification of nascent translation within cercarial heads and tails shows increased levels in cercarial tails. Normalized puromycin signal for heads only and tails only from confocal images. Average puromycin treated signal intensity was normalized by the average DAPI intensity after removal of the untreated background signal. Heads only was then set to zero. Heads only (n = 11) and tails only (n = 8). Tails only treated (n = 8) was compared against tails only untreated (n = 7). P-values *** = ≤ 0.001 ** = ≤ 0.01. The statistical significance was calculated using the Mann-Whitney and an unpaired t-test.

To verify these initial observations, we used immunohistochemistry and confocal imaging to localize active translation in cercarial heads and cercarial tails (Fig 2). We found a stark contrast in protein expression (seen in green) between the cercarial tails and heads. Image quantification confirmed that the puromycin signal in heads is nearly undetected above background (Fig 3) supporting our Western blot analysis. In fact, it is clear that translation primarily occurs in cercarial tails, and is mostly absent in heads by IHC. Thus, the majority of translation occurs in tails. Representative images of untreated cercariae can be seen in supplement (S1 Fig). We used DAPI normalization to estimate the number of nucleated cells in cercarial heads and tails. The average DAPI signal intensity from heads had a 2 to 1 ratio of nucleated cells over cercarial tails (S2 Fig). The estimate for cell counts corresponds to the signal change when the amount of protein used for whole cercariae in (Fig 1) required a 2 to 1 increase from5from 5 μg to 10 μg to match the signal of tails alone at 5 μg.

### Nascent translation rate increase as schistosomula develop

**Fig 4:**
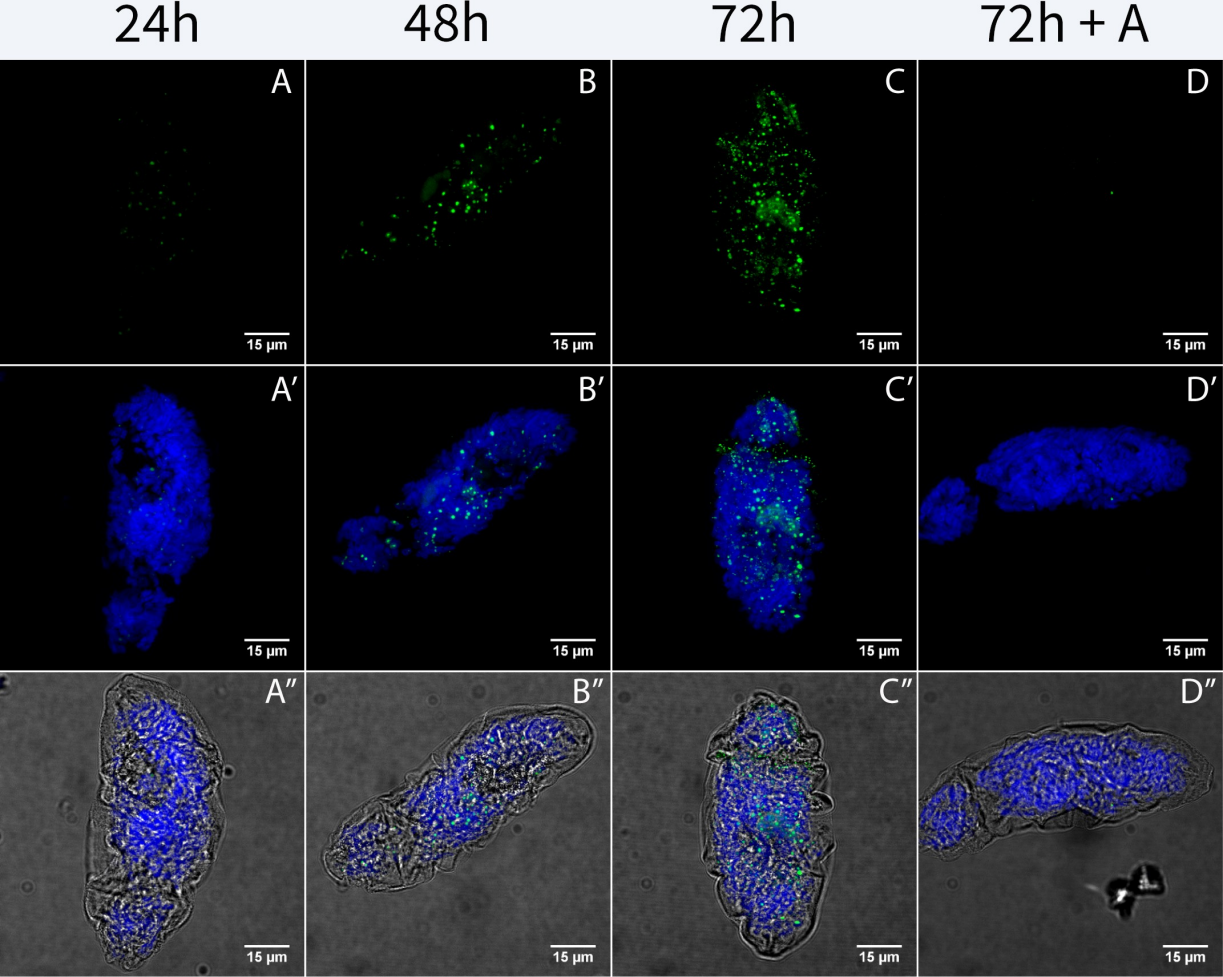
Nascent translation accelerates over the first 72 hours of schistosomula development. Confocal maximum projections of puromycylated schistosomula over 72-hour time course. Anti- Puromycin PMY-2A4 primary with Alexa 647 secondary (+P) was used to detect nascent translation. The competitive translational inhibitor anisomycin (+A) was used to verify the specificity of the puromycin treatment. Sample are: 24-hour schistosomulum (A, A’, A”), 48-hour schistosomulum (B, B’, B”), 72-hour schistosomulum (C, C’, C”), and 72- hour schistosomulum with the anisomycin competition (D, D’, D”). Samples in Row 1 are labeled to visualize puromycin labeling in green (A-D), samples in Row 2 are labeled to visualize puromycin in green and DAPI in blue (A’-D’), and samples in Row 3 are labeled to visualize puromycin in green, DAPI in blue and the bright field overlay (A” -D”). Background correction was applied using Image J.

Schistosomula have a large burst of transcription that is highest at 3–4 hours after transformation and continues through the first 24 hours post-transformation [5]. The translation rate shows a continual increase over a 24 hour period [7]. To further explore the changes in global translation levels during early schistosome development we measured translation activity in schistosomula during the initial 72 hours after transformation. We collected schistosomula at 24- hour increments over 3 days and visualized translation by immunohistochemistry using an Alexa 647 secondary antibody (Fig 4A–4C). For each time point, schistosomula were puromycylated 30 minutes prior to sample collection and fixation in order to get snapshots of active nascent translation. While 4-hour schistosomula showed little detectable translation (Fig 1), puromycylated schistosomula at later stages had nascent translation increases for the first 72 hours of development *in vitro* (Fig 4) compared to untreated individuals (S3 Fig). In order to test the validity of the puromycylation results and verify that puromycin actively targeted ribosomes and active transcripts, we pretreated samples for 20 minutes with the drug anisomycin. Anisomycin competes with puromycin for binding to the ribosomal A-site. Expectedly, anisomycin should block puromycin from binding to active transcripts resulting in loss of the puromycin signal. We observed that following a 20 minute pretreatment with anisomycin, 72-hour schistosomula were almost devoid of the puromycylation signal demonstrating that puromycin specifically targets nascent peptides (Fig 4D–4D”).

### Translational inhibitors translational inhibitors preferentially inhibit motility at high concentration

**Fig 5:**
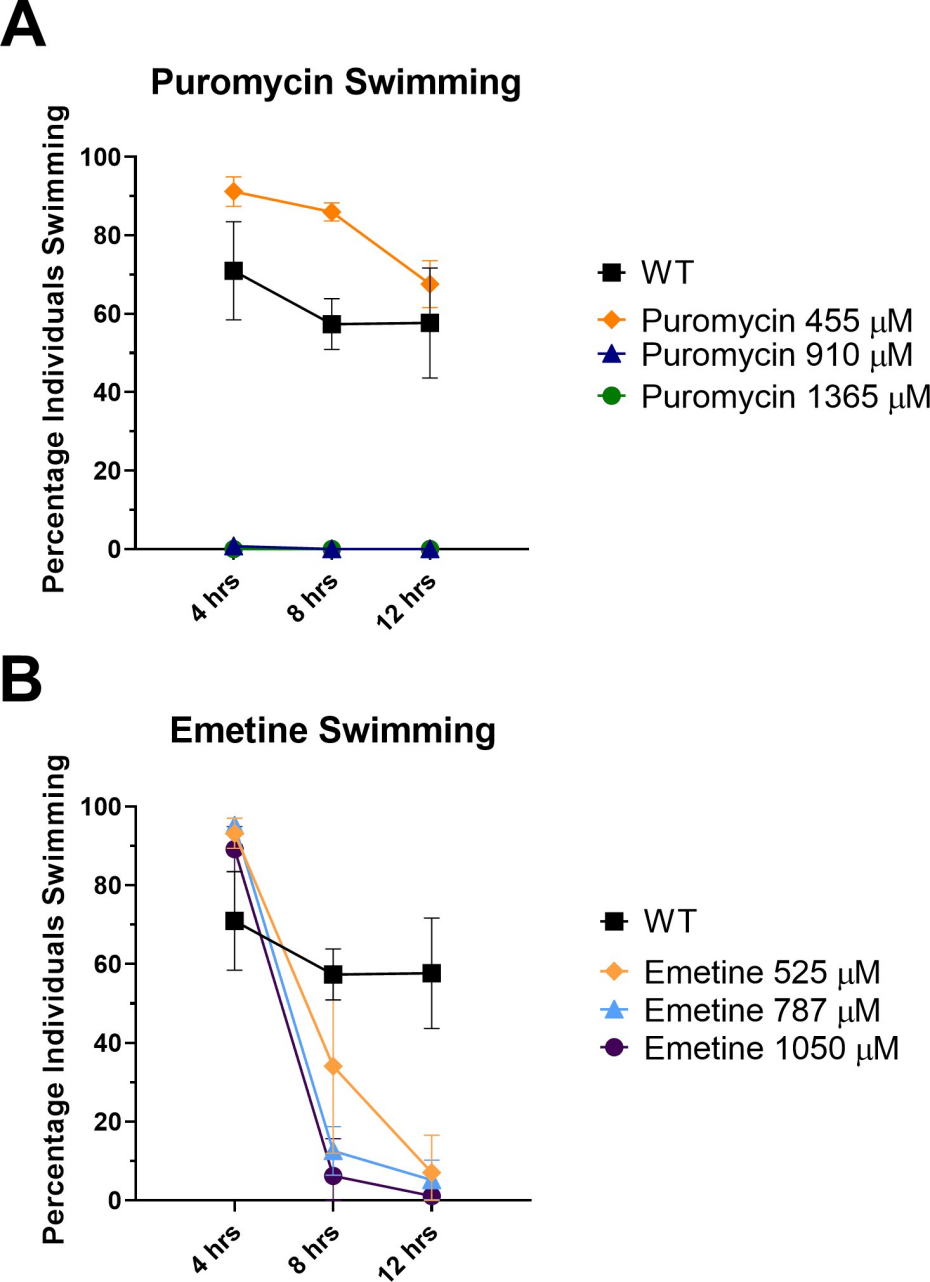
Effects of translational inhibitors on cercarial motility. Summary quantitation of swimming individuals from population. Cercariae (n = ~75) per well were cultured in 96-well plates for 12 hours after treatment with translational inhibitors. Translational inhibitors emetine and puromycin were given at the following concentrations: 525 μM, 787 μM, and 1050 μM emetine and 455 μM, 910 μM, and 1365 μM puromycin. Wild Type (WT) negative control was untreated. (A) shows the percentage of emetine treated individuals swimming in the water column at each time point 4, 8, and 12 hours. (B) shows the percentage of puromycin treated individuals swimming in the water column at each time point 4, 8, and 12 hours. Wild type negative control samples are shown swimming (A, B). The statistical significance was calculated using one-way ANOVA with Dunnett’s multiple comparison against untreated samples.

We further explored whether translation is important in cercarial tails and tested if repression of this translation could negatively affect cercarial behavior and viability. To address this, we exposed cercariae to translational inhibitors for 12 hours at 16° C (Fig 5), expanding on previous reports that showed that translational inhibitors have little effect on cercarial transformation and tegumental remodeling [25]. We quantitatively assessed motility and viability in the presence of high concentrations of inhibitors of translation. We exposed cercariae to the translational inhibitors puromycin (Fig 5A) and emetine (Fig 5B), each of which has been shown to inhibit translation in other schistosome life stages [6, 25, 26]. Negative control samples were left untreated (Fig 5). After inhibition of translation for 4 hour at 16°C, we observed no change in swimming behavior in the emetine treated individuals at all concentrations compared to the wild type (Fig 5B). The puromycin treated individuals at 455 μM concentration also showed no change in swimming after 4 hours of treatment (Fig 5A). At higher concentrations 910 μM and 1365 μM puromycin showed complete ablation of swimming after 4 hours (Fig 5A). After inhibition of translation for 8 hours at 16° C, the emetine treated samples at all concentrations showed significantly reduced swimming capability compared to the wild type (Fig 5B). Emetine and puromycin have both been shown to be effective translational inhibitors in schistosomes at the concentrations used [14]. The cercariae treated at 455 μM puromycin showed an increase in swimming behavior up to 8 hours after treatment began (Fig 5A), this led us to assay the effects of low levels of translational inhibitors on cercarial longevity.

### Translational inhibitors increase cercarial longevity at low concentration

**Fig 6:**
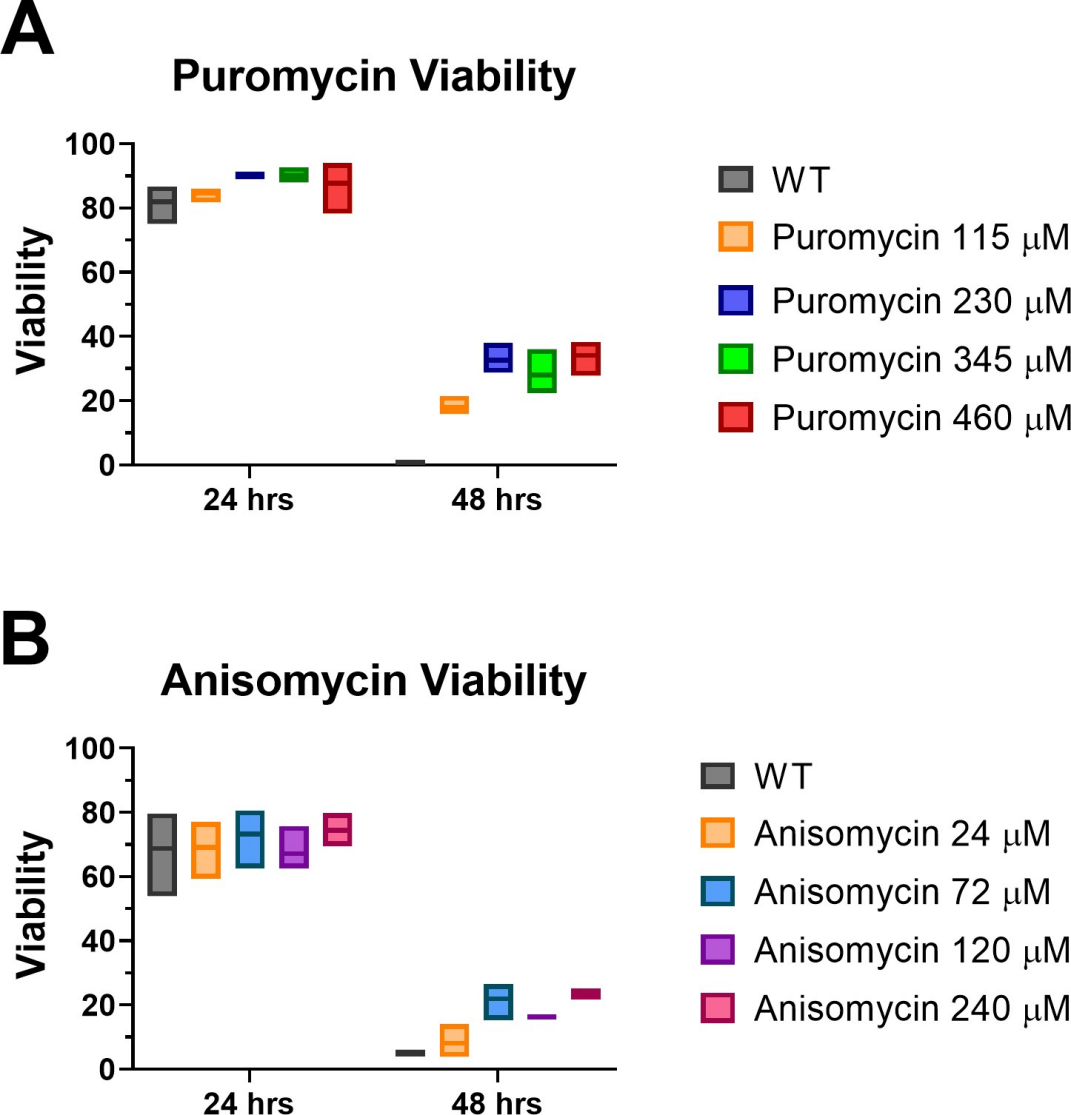
Effects of translational inhibitors on cercarial longevity. Summary quantitation of viable individuals from population. Cercariae (n = ~75) were cultured in 96-well plates for 48 hours after treatment with translational inhibitors. Viability was determined using propidium iodide staining. Translational inhibitors anisomycin and puromycin were given at the following concentrations: 24 μM, 72 μM, 120 μM, and 240 μM anisomycin and 115 μM, 230 μM, 345 μM, and 460 μM puromycin. Wild Type (WT) negative control was untreated. (A) shows viability of anisomycin treated individuals at 24 and 48 hours post treatment. (B) shows viability of puromycin treated individuals at each time point 24 and 48 hours post treatment. All treatments including WT were performed in triplicate. The statistical significance was calculated using one-way ANOVA with Dunnett’s multiple comparison against untreated samples.

The translational inhibitors tested showed a marked inhibition of swimming behavior at high concentrations (~1mM). The opposite effect was observed at the lower concentrations for puromycin, showing a significant increase in number of individuals swimming 4 and 8 hours after treatment began. This led us to explore the effects of lower levels of translational inhibitors on longevity. Other systems have shown that a low level global reduction of translation can both reduce energy consumption and increase life span [27, 28]. This phenomenon has not been explored in schistosomes and specifically not within the short lived cercarial stage. Cercariae were treated with low levels of anisomycin and puromycin and observed over a 48 hour period using propidium iodide as a viability stain. After 24 hours at 16° C all samples showed significant viability for both the anisomycin and puromycin treated groups as well as their respective wild type controls (Fig 6A and 6B). The wild type samples do not survive well at the 48 hour time point showing ~5% viability among both untreated groups (Fig 6). Puromycin shows a significant increase in viable individuals at all treatment levels after 48 hours with a range of 17–34% viability (Fig 6A). Anisomycin was less effective but still showed significantly increased viability at 48 hours for the samples treated at 72 μM and 240 μM compared to wild type (Fig 6B). Both 72 μM and 240 μM anisomycin averaged ~22% viability at 48 hours post treatment. The samples treated at 120 μM were not significantly increased above wild type but were approaching significance.

## Discussion and conclusion

In this manuscript, we made four critical observations. First, we show translation levels are distinctly different in schistosome larval structures- the cercarial head and the cercarial tail, with higher levels of translation in cercarial tails and limited translational activity in cercarial heads. Second, we provide data that transcription and translation in cercariae are decoupled. Third, we report that this decoupled transcription and translation continues into the schistosomula stage, but translation levels increase over 72 hours. Finally, we find that translational repression in the cercarial stage has varying behavioral and biological effects depending on level of drug treatment; high levels of inhibitors lead to attenuated swimming behavior and low concentrations of inhibitors lead to an increase in longevity. Our findings support a previous study that reports translation levels are reduced in schistosomula [7]. We were initially interested in the rates of protein translation in schistosomula. We found that there were consistently low levels of translation products identified when expressing heterologous genes in schistosomes, even though high transcriptional products were readily available [6]. This discrepancy between transcript levels and protein level led us to analyze the levels of translation in cercariae and in schistosomula. Consequently, we attempted to address several questions of interest: 1) If cercariae do not transcribe new RNA [5], do cercariae translate new proteins; if so 2), are there differences in translation between cercarial macrostructures, the head and the tail? 3) Are transcription and translation differentially regulated in schistosomula? In order to measure translation levels, we adapted the Sunset and Punch-P puromycylation labeling systems to work with *S*. *mansoni* [22, 29].

After a cercaria exits the snail host, it swims to initiate interaction with a definitive human host using its tail. When it makes contact with the mammalian host, the cercaria initiates host invasion that includes the use of the tail and proteases to penetrate the skin and the eventual loss of the cercarial tail. The transformed cercarial head then continues to develop independently of the tail, and develops into an adult worm over several weeks. The tail is no longer used for development. Nearly all published transcriptomic studies that incorporate the cercarial larval stage, including our own published work, have analyzed and focused on the cercariae as a whole entity, extracting total cercarial RNA and extrapolating from these data that the cercaria primarily swim to invade the next host and contain proteases for host invasion [4, 11, 20, 26, 30–33]. The data we present here suggests that while this is partially correct, this approach is not sufficient for identification of the molecular requirements for continued development in the mammalian host. After cercariae exit the snail, they do not undergo new transcription corroborating the well-accepted idea that all transcripts for translation are already prepackaged prior to exit from the molluscan host [5]. However, protein translation occurs readily but unequally in the cercarial head and tail (Fig 1). While translation is high in the actively mobile tails, translation levels are significantly limited in cercarial heads (Figs 1–3), which continue development into schistosomula and eventually into adult worms. The mechanism for how cercariae can have two completely distinct translational programs for the head and tail is not clear, nor is it obvious what role repressed translation in cercarial heads has on further development. Presumably, the tail requires translation of the metabolic genes in order to maintain the energy to maintain robust swimming activity [11, 20, 32, 34, 35], whereas the cercarial head remains quiescent to conserve the limited energy needed when a host is identified and invaded.

We also tested whether the translation in the cercarial tail is essential by using established translational inhibitors to determine if they could prevent cercarial mobility. The addition of high concentrations of translational inhibitors had significant effect on cercarial motility over a 12-hour period (Fig 5). The treatment showed a preferential effect on motility before an effect on viability was seen (Figs 5 and S4). Both emetine and puromycin at concentrations that have been shown to significantly inhibit translation in schistosomes showed this pattern of motility being attenuated prior to any decrease in viability [14, 25, 36]. This preference suggests that the cercarial tail which is required for motility is more directly affected than the cercarial head which is necessary to progress to the next life stage. This is in support of work by others showing that the use of inhibitors, such as puromycin, did not prevent cercarial transformation into schistosomula [36]. High concentrations of translational inhibitors show a significant effect on swimming but we found that at lower concentrations swimming behavior was increased over the 12 hour observation period. We then wanted to determine if this effect not only altered swimming but led to a change in overall longevity as seen in *C*. *elegans*, mice, and yeast [27, 28, 37].

Treatment with low levels of translational inhibitors not only showed an increase in swimming behavior, which is likely attributed to a reduction of energy consumption from reduced translation [38], but also showed a significant increase in longevity above that of the untreated wild type individuals (Fig 6). The likely mechanisms reported in other systems are the unfolded protein response pathway, the global stress response pathways, and autophagy [27, 28, 37, 39]. Given the preliminary and unexpected nature of our findings it is unclear which is the most likely within our system. Cercariae experience significant stressors as they transition to the schistosomula stage and this suggests the poised nature of their response machinery. Cercariae also lack a source of significant new resource uptake from fresh water. They have shown increased longevity under reduced temperature as well as in the presence of glucose [12, 40]. These data together support a potential increase in longevity based on energy utilization. Given the consumptive nature of protein synthesis this is the most likely given our current understanding of cercarial longevity and its connection to translational regulation.

In addition to differential regulation of translation between cercarial heads and tails, the schistosomula maintain limited translation during a period when high levels of transcription occur [5]. Translation levels gradually increase over 72 hours *in vitro* during a time when there is significant transcription (Fig 4). Given our current understanding of the development of *in vivo* and *in vitro* derived schistosomula it is likely the pattern observed holds true in both groups. Ultra-structure analysis via microscopy shows similar patterns of development though they occur on differing time scales [41]. Transcriptional analysis has also shown that skin derived compared to mechanically transformed schistosomula show similar expression profiles [42]. Thus, transcription and translation are decoupled in the schistosomula. It appears that the cercarial head transports a subset of proteins that function with limited new translation after the tail is lost and that these proteins are sufficient for continued survival. Identifying the initial proteins in cercarial heads using mass spectrometry could elucidate potential functional roles in the developing schistosomula. However, the extensive number of proteins and proteases found in the cercarial glands used for invasion including various proteases [43–45] could present technical challenges and false positives that would need to be excluded.

There are several possible mechanisms for translational repression in cercarial heads and schistosomula. Two likely modes of repression are a lack of ribosomal biogenesis or cap-dependent translational repression [46–48]. The levels of ribosomal biogenesis can be assessed by measuring the levels of rRNA processing during development. It is clear that some peptides are being produced as they are detectable via western blot, but they represent a significant decrease compared to the tail tissue which has less mass and significantly fewer cells per individual (Figs 1 and S3). Cercarial heads and early schistosomula could also potentially initiate cap-independent translational initiation, which could allow for gene-specific upregulation during a period of global repression as seen in some stress and other signaling responses [49]. These potential internal ribosomal entry sites commonly require partner proteins that would give insight into the language of translational regulation within schistosomes [50].

Schistosome could also make use of regulatory RNAs as another potential mechanism for translational control. Recently, a long series of both microRNAs and long non-coding RNA have been identified in schistosomes [51–56]. Both microRNA (miRNAs) and long non-coding RNAs (lncRNAs) have been implicated in translational repression [57–59], and decoupled transcription and translation have been implicated in aging [60]. A role for schistosome regulatory RNAs is only recently being explored, but could have positive implications for our understanding of schistosome development.

The cercarial head is necessary and sufficient to proceed to the schistosomula stage. However, this does not negate the need to understand transcription and translation within the tail region [61]. Differential regulation in cercarial protein translation is of interest and could give us clues into the requirements for schistosome development after host invasion, and it provide novel insights into general biological mechanisms of translational control. After cercariae transform into the schistosomula, translation levels, while differentially regulated from transcription, gradually increase over 72 hours (Fig 4). The identification of the protein products and the timeline of their production in cercarial heads and schistosomula could further provide insight into the processes schistosomes use to adapt to the host environment and develop into adult worms. Further, a comparative analysis of both the transcriptome and proteome of the cercarial head could identify critical prepackaged transcripts and proteins involved in parasite survival and development for use after host invasion. This potential analysis could provide targets for early genetic manipulation and for the expression of heterologous genes when translation is normally suppressed.

## Supporting information

S1 Fig Representative confocal images of untreated cercariae. Untreated *S*. *mansoni* cercariae used for background correction of heads vs tails quantitation. Representative max projections from puromycin untreated samples. Image J used for ROI and intensity analysis. No adjustments or background correction performed on these images. Puromycin staining in green and DAPI stain in blue. (A-D) intact cercaria; A puromycin signal alone, B DAPI signal alone, C DAPI and puromycin overlay, D DAPI, puromycin, and bright-field overlay. (E-H) cercarial head alone; E puromycin signal alone, F DAPI signal alone, G DAPI and puromycin overlay, H DAPI, puromycin, and bright-field overlay. (I-L) cercarial tail alone; I puromycin signal alone, J DAPI signal alone, K DAPI and puromycin overlay, L DAPI, puromycin, and bright-field overlay.
(TIF)

S2 Fig Average DAPI intensity heads vs tails. Graph of average DAPI intensity of max projections from puromycin treated and untreated cercariae. ROI and intensity analysis performed using image J. Statistical analysis using Mann U Whitney test. *** = P-value ≤≤ 0.001.
(TIFF)

S3 Fig Representative confocal images of untreated schistosomula. Untreated *S*. *mansoni* schistosomula images from 24 hours, 48 hours, and 72 hours post-transformation shown as representative max projections. (A-A”) 24 hour untreated schistomulum, (B-B”) 48 hour untreated schistosomulum, (C-C”) 72 hour untreated schistosomulum. (A, B, C) puromycin signal, (A’, B’, C’) DAPI signal, and (A”, B”, C”) bright-field image. Image processing performed using ImageJ.
(TIF)

S4 Fig Puromycin and emetine viability high concentration 12 hour time course. Summary quantitation of viable individuals from population. Cercariae (n = ~75) were cultured in 96-well plates for 12 hours after treatment with translational inhibitors. Viability was determined using propidium iodide staining. Translational inhibitors emetine and puromycin were given at the following concentrations: 525 μM, 787 μM, and 1050 μM emetine and 455 μM, 910 μM, and 1365 μM puromycin. Wild Type (WT) negative control was untreated. (A) shows viability of puromycin treated individuals at 4, 8, and 12 hours post treatment. (B) shows viability of emetine treated individuals at each time point 4, 8, and 12 hours post treatment. All treatments including wildtype were performed in triplicate.
(TIF)
