# Supplementary figures and images for: Heads or tails? Differential translational regulation in cercarial heads and tails of schistosome worms
Source: PLoS One. 2019 Oct 28;14(10):e0224358. doi: 10.1371/journal.pone.0224358 (PMC6816793; doi:10.1371/journal.pone.0224358)

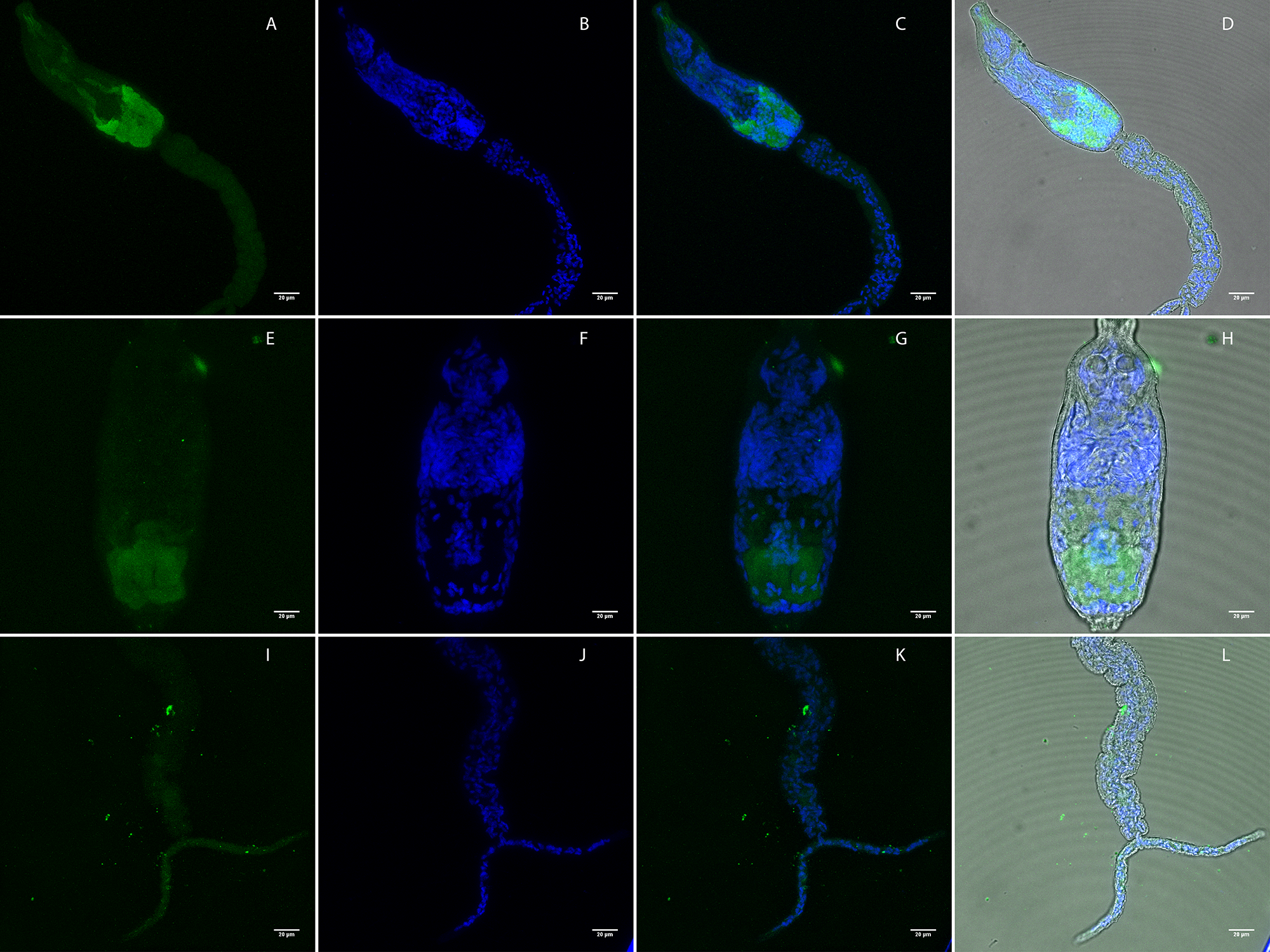

Supplement: S1 Fig — Untreated S. mansoni cercariae used for background correction of heads vs tails quantitation. Representative max projections from puromycin untreated samples. Image J used for ROI and intensity analysis. No adjustments or background correction performed on these images. Puromycin staining in green and DAPI stain in blue. (A-D) intact cercaria; A puromycin signal alone, B DAPI signal alone, C DAPI and puromycin overlay, D DAPI, puromycin, and bright-field overlay. (E-H) cercarial head alone; E puromycin signal alone, F DAPI signal alone, G DAPI and puromycin overlay, H DAPI, puromycin, and bright-field overlay. (I-L) cercarial tail alone; I puromycin signal alone, J DAPI signal alone, K DAPI and puromycin overlay, L DAPI, puromycin, and bright-field overlay. (TIF) [file pone.0224358.s001.tif]

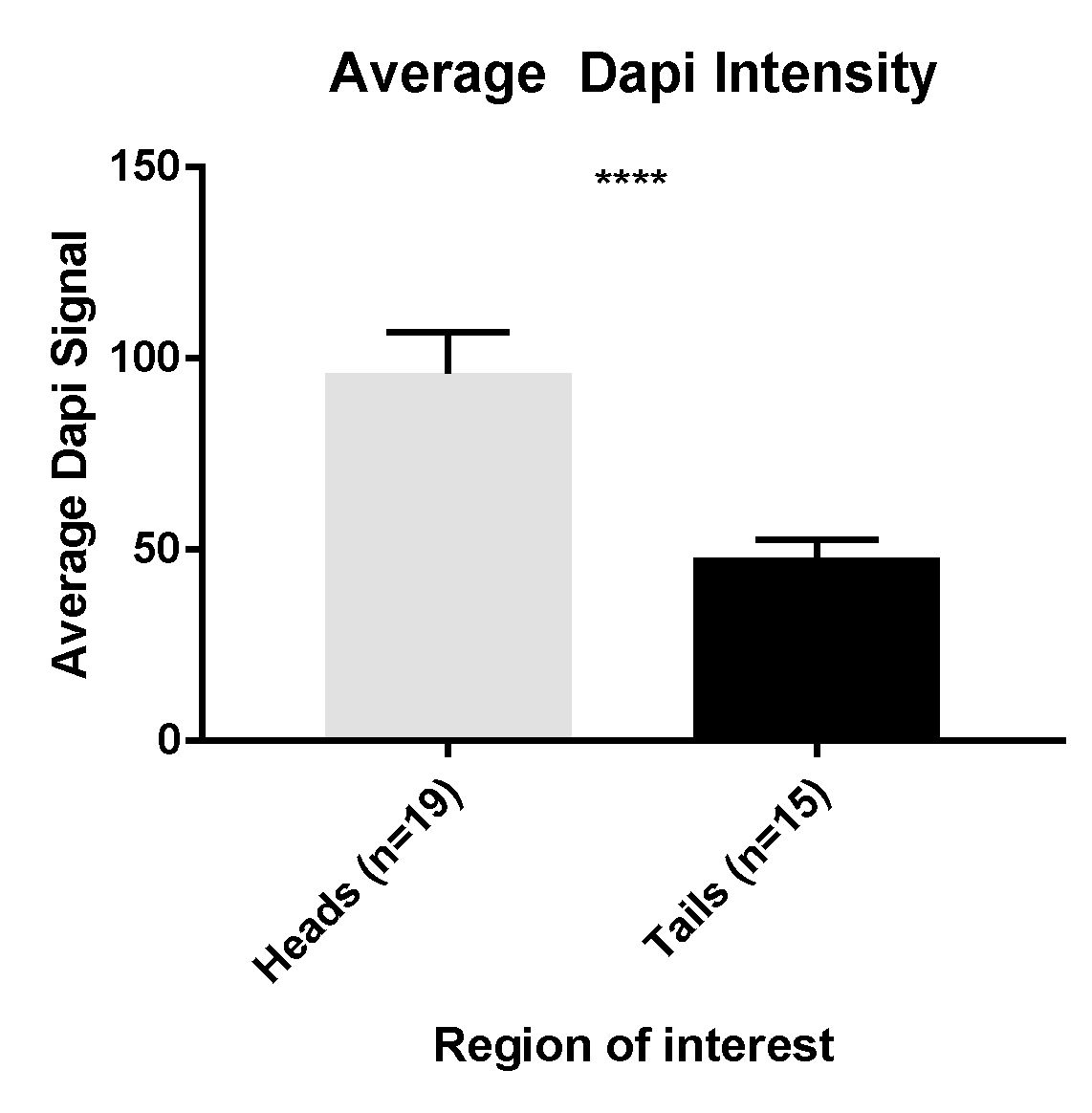

Supplement: S2 Fig — Graph of average DAPI intensity of max projections from puromycin treated and untreated cercariae. ROI and intensity analysis performed using image J. Statistical analysis using Mann U Whitney test. *** = P-value ≤≤ 0.001. (TIFF) [file pone.0224358.s002.tiff]

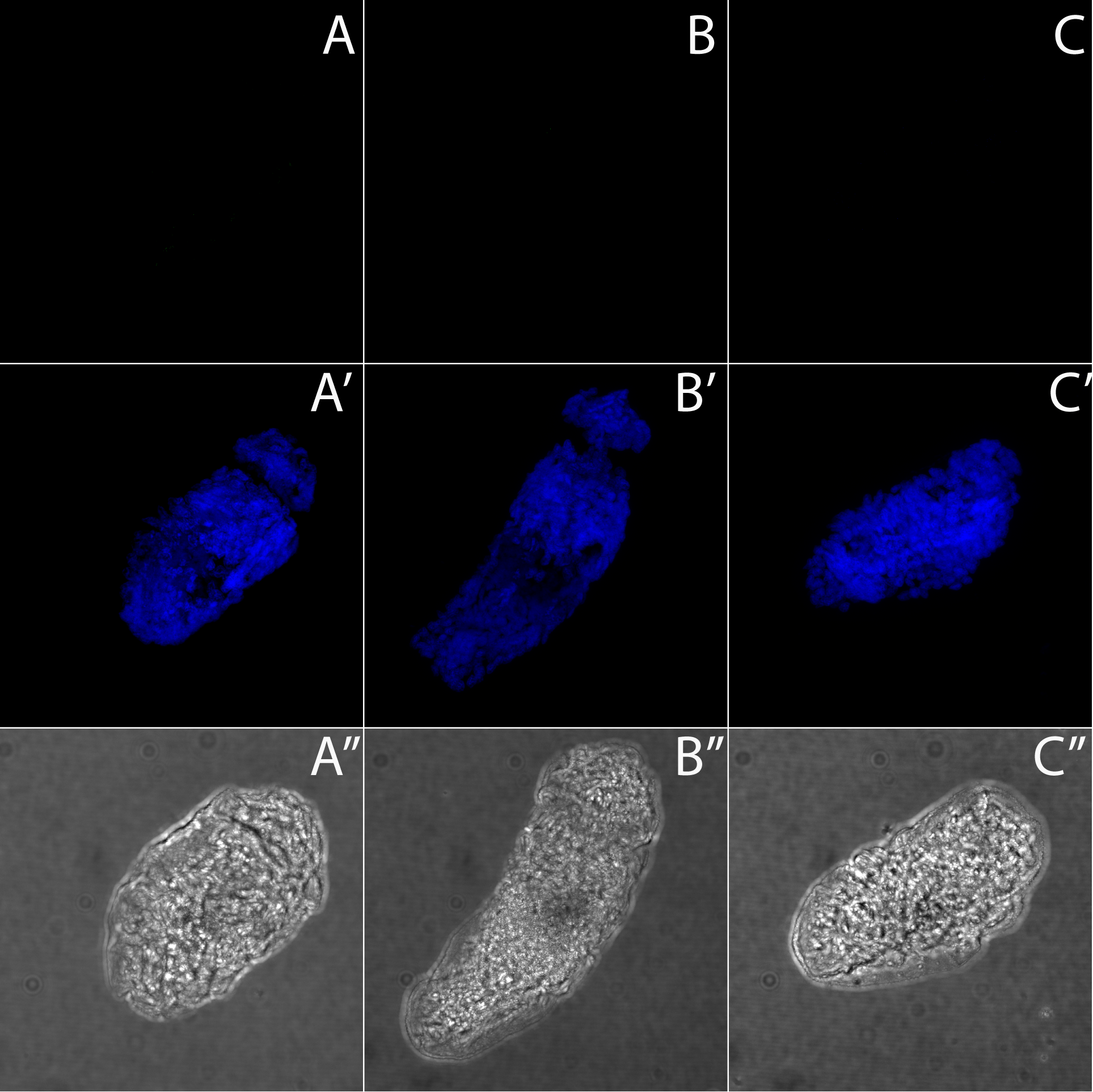

Supplement: S3 Fig — Untreated S. mansoni schistosomula images from 24 hours, 48 hours, and 72 hours post-transformation shown as representative max projections. (A-A”) 24 hour untreated schistomulum, (B-B”) 48 hour untreated schistosomulum, (C-C”) 72 hour untreated schistosomulum. (A, B, C) puromycin signal, (A’, B’, C’) DAPI signal, and (A”, B”, C”) bright-field image. Image processing performed using ImageJ. (TIF) [file pone.0224358.s003.tif]
